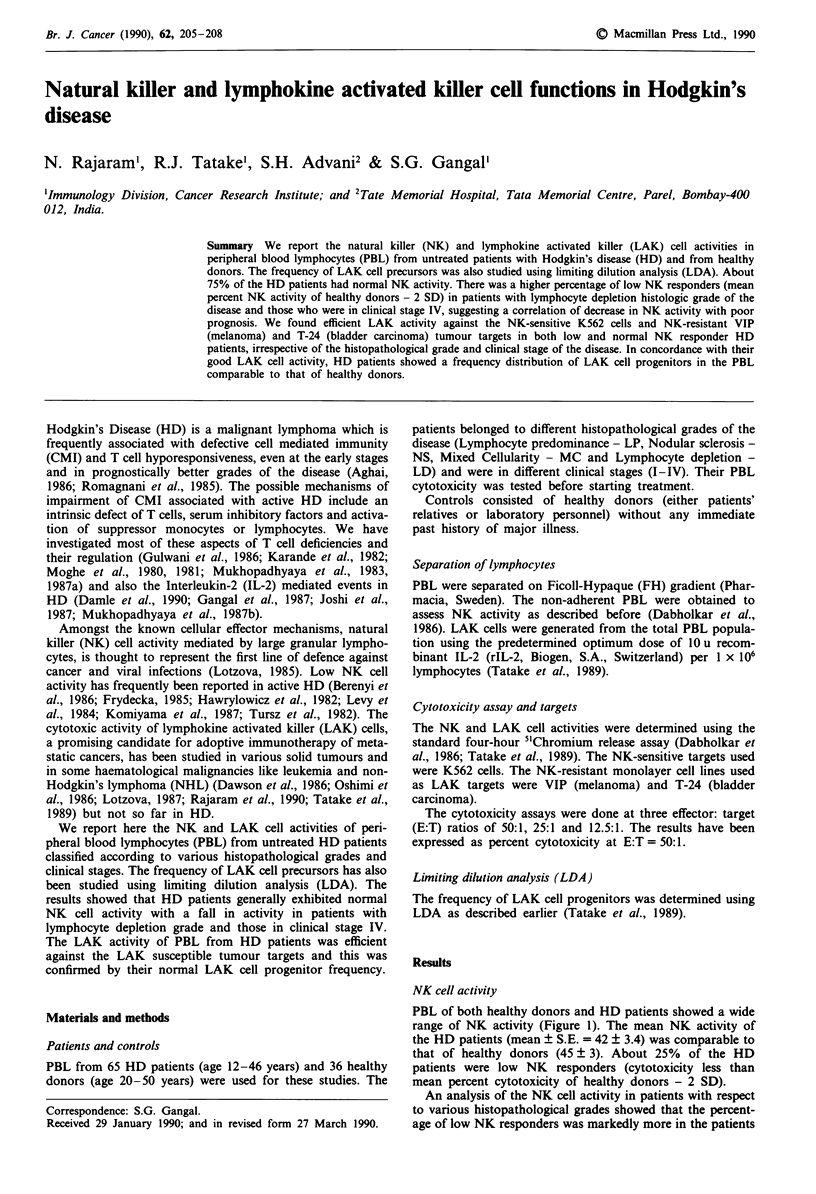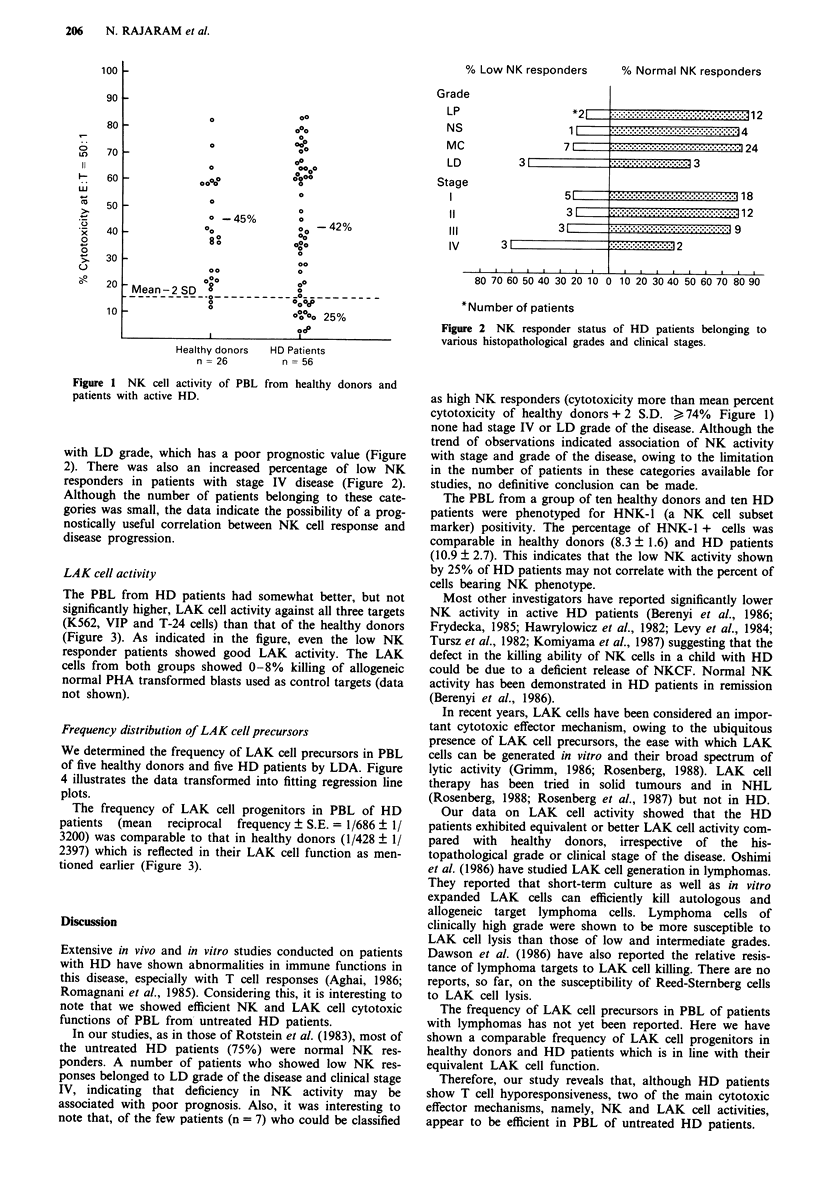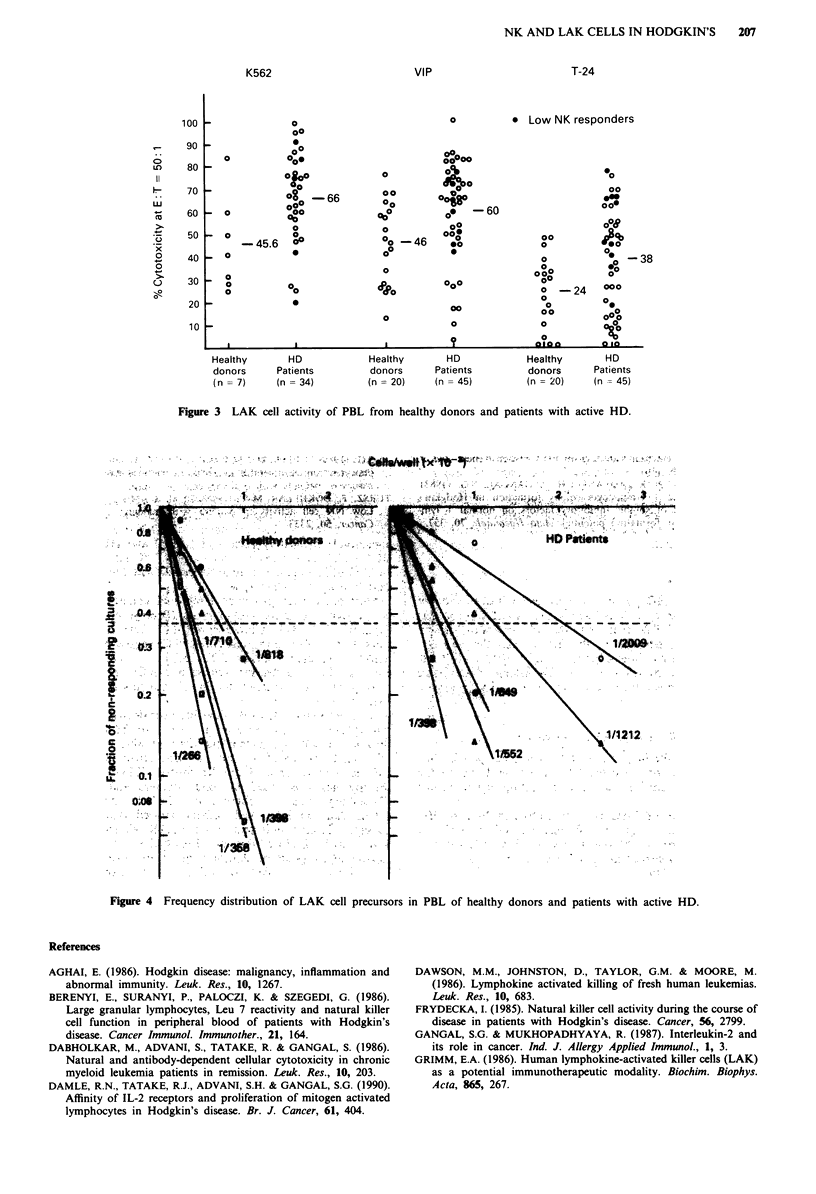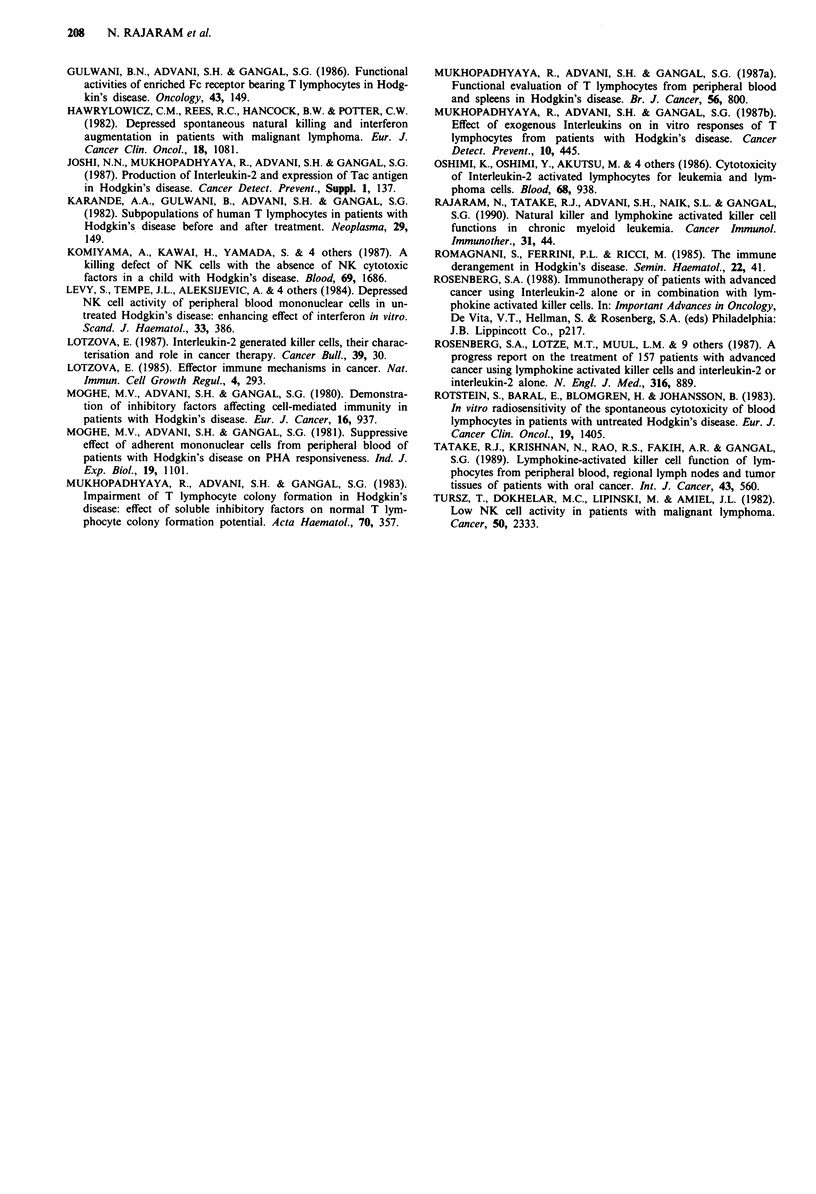# Natural killer and lymphokine activated killer cell functions in Hodgkin's disease.

**DOI:** 10.1038/bjc.1990.261

**Published:** 1990-08

**Authors:** N. Rajaram, R. J. Tatake, S. H. Advani, S. G. Gangal

**Affiliations:** Immunology Division, Cancer Research Institute, Parel, Bombay, India.

## Abstract

We report the natural killer (NK) and lymphokine activated killer (LAK) cell activities in peripheral blood lymphocytes (PBL) from untreated patients with Hodgkin's disease (HD) and from healthy donors. The frequency of LAK cell precursors was also studied using limiting dilution analysis (LDA). About 75% of the HD patients had normal NK activity. There was a higher percentage of low NK responders (mean percent NK activity of healthy donors--2 SD) in patients with lymphocyte depletion histologic grade of the disease and those who were in clinical stage IV, suggesting a correlation of decrease in NK activity with poor prognosis. We found efficient LAK activity against the NK-sensitive K562 cells and NK-resistant VIP (melanoma) and T-24 (bladder carcinoma) tumour targets in both low and normal NK responder HD patients, irrespective of the histopathological grade and clinical stage of the disease. In concordance with their good LAK cell activity, HD patients showed a frequency distribution of LAK cell progenitors in the PBL comparable to that of healthy donors.


Br. J. Cancer (1990), 62, 205-208 © Macmillan Press Ltd., 1990

### Natural killer and lymphokine activated killer cell functions in Hodgkin's disease

N. Rajaram[1], R.J. Tatake[1], S.H. Advani[2] & S.G. Gangal[1]

[1]*Immunology Division, Cancer Research Institute; and* [2]*Tate Memorial Hospital, Tata Memorial Centre, Parel, Bombay-400 012, India.*

**Summary** We report the natural killer (NK) and lymphokine activated killer (LAK) cell activities in peripheral blood lymphocytes (PBL) from untreated patients with Hodgkin's disease (HD) and from healthy donors. The frequency of LAK cell precursors was also studied using limiting dilution analysis (LDA). About 75% of the HD patients had normal NK activity. There was a higher percentage of low NK responders (mean percent NK activity of healthy donors - 2 SD) in patients with lymphocyte depletion histologic grade of the disease and those who were in clinical stage IV, suggesting a correlation of decrease in NK activity with poor prognosis. We found efficient LAK activity against the NK-sensitive K562 cells and NK-resistant VIP (melanoma) and T-24 (bladder carcinoma) tumour targets in both low and normal NK responder HD patients, irrespective of the histopathological grade and clinical stage of the disease. In concordance with their good LAK cell activity, HD patients showed a frequency distribution of LAK cell progenitors in the PBL comparable to that of healthy donors.

Hodgkin's Disease (HD) is a malignant lymphoma which is frequently associated with defective cell mediated immunity (CMI) and T cell hyporesponsiveness, even at the early stages and in prognostically better grades of the disease (Aghai, 1986; Romagnani *et al.*, 1985). The possible mechanisms of impairment of CMI associated with active HD include an intrinsic defect of T cells, serum inhibitory factors and activation of suppressor monocytes or lymphocytes. We have investigated most of these aspects of T cell deficiencies and their regulation (Gulwani *et al.*, 1986; Karande *et al.*, 1982; Moghe *et al.*, 1980, 1981; Mukhopadhyaya *et al.*, 1983, 1987a) and also the Interleukin-2 (IL-2) mediated events in HD (Damle *et al.*, 1990; Gangal *et al.*, 1987; Joshi *et al.*, 1987; Mukhopadhyaya *et al.*, 1987b).

Amongst the known cellular effector mechanisms, natural killer (NK) cell activity mediated by large granular lymphocytes, is thought to represent the first line of defence against cancer and viral infections (Lotzova, 1985). Low NK cell activity has frequently been reported in active HD (Berenyi *et al.*, 1986; Frydecka, 1985; Hawrylowicz *et al.*, 1982; Levy *et al.*, 1984; Komiyama *et al.*, 1987; Tursz *et al.*, 1982). The cytotoxic activity of lymphokine activated killer (LAK) cells, a promising candidate for adoptive immunotherapy of metastatic cancers, has been studied in various solid tumours and in some haematological malignancies like leukemia and non-Hodgkin's lymphoma (NHL) (Dawson *et al.*, 1986; Oshimi *et al.*, 1986; Lotzova, 1987; Rajaram *et al.*, 1990; Tatake *et al.*, 1989) but not so far in HD.

We report here the NK and LAK cell activities of peripheral blood lymphocytes (PBL) from untreated HD patients classified according to various histopathological grades and clinical stages. The frequency of LAK cell precursors has also been studied using limiting dilution analysis (LDA). The results showed that HD patients generally exhibited normal NK cell activity with a fall in activity in patients with lymphocyte depletion grade and those in clinical stage IV. The LAK activity of PBL from HD patients was efficient against the LAK susceptible tumour targets and this was confirmed by their normal LAK cell progenitor frequency.

### Materials and methods

### *Patients and controls*

PBL from 65 HD patients (age 12-46 years) and 36 healthy donors (age 20-50 years) were used for these studies. The patients belonged to different histopathological grades of the disease (Lymphocyte predominance - LP, Nodular sclerosis - NS, Mixed Cellularity - MC and Lymphocyte depletion - LD) and were in different clinical stages (I-IV). Their PBL cytotoxicity was tested before starting treatment.

Controls consisted of healthy donors (either patients' relatives or laboratory personnel) without any immediate past history of major illness.

### *Separation of lymphocytes*

PBL were separated on Ficoll-Hypaque (FH) gradient (Pharmacia, Sweden). The non-adherent PBL were obtained to assess NK activity as described before (Dabholkar *et al.*, 1986). LAK cells were generated from the total PBL population using the predetermined optimum dose of 10 u recombinant IL-2 (rIL-2, Biogen, S.A., Switzerland) per $1 \times 10^6$ lymphocytes (Tatake *et al.*, 1989).

### *Cytotoxicity assay and targets*

The NK and LAK cell activities were determined using the standard four-hour $^{51}$Chromium release assay (Dabholkar *et al.*, 1986; Tatake *et al.*, 1989). The NK-sensitive targets used were K562 cells. The NK-resistant monolayer cell lines used as LAK targets were VIP (melanoma) and T-24 (bladder carcinoma).

The cytotoxicity assays were done at three effector: target (E:T) ratios of 50:1, 25:1 and 12.5:1. The results have been expressed as percent cytotoxicity at E:T = 50:1.

### *Limiting dilution analysis (LDA)*

The frequency of LAK cell progenitors was determined using LDA as described earlier (Tatake *et al.*, 1989).

### Results

### *NK cell activity*

PBL of both healthy donors and HD patients showed a wide range of NK activity (Figure 1). The mean NK activity of the HD patients (mean ± S.E. = 42 ± 3.4) was comparable to that of healthy donors (45 ± 3). About 25% of the HD patients were low NK responders (cytotoxicity less than mean percent cytotoxicity of healthy donors - 2 SD).

An analysis of the NK cell activity in patients with respect to various histopathological grades showed that the percentage of low NK responders was markedly more in the patients

Correspondence: S.G. Gangal.
Received 29 January 1990; and in revised form 27 March 1990.

**Figure 1** NK cell activity of PBL from healthy donors and patients with active HD.

with LD grade, which has a poor prognostic value (Figure 2). There was also an increased percentage of low NK responders in patients with stage IV disease (Figure 2). Although the number of patients belonging to these categories was small, the data indicate the possibility of a prognostically useful correlation between NK cell response and disease progression.

### *LAK cell activity*

The PBL from HD patients had somewhat better, but not significantly higher, LAK cell activity against all three targets (K562, VIP and T-24 cells) than that of the healthy donors (Figure 3). As indicated in the figure, even the low NK responder patients showed good LAK activity. The LAK cells from both groups showed 0–8% killing of allogeneic normal PHA transformed blasts used as control targets (data not shown).

### *Frequency distribution of LAK cell precursors*

We determined the frequency of LAK cell precursors in PBL of five healthy donors and five HD patients by LDA. Figure 4 illustrates the data transformed into fitting regression line plots.

The frequency of LAK cell progenitors in PBL of HD patients (mean reciprocal frequency ± S.E. = 1/686 ± 1/3200) was comparable to that in healthy donors (1/428 ± 1/2397) which is reflected in their LAK cell function as mentioned earlier (Figure 3).

### Discussion

Extensive *in vivo* and *in vitro* studies conducted on patients with HD have shown abnormalities in immune functions in this disease, especially with T cell responses (Aghai, 1986; Romagnani *et al.*, 1985). Considering this, it is interesting to note that we showed efficient NK and LAK cell cytotoxic functions of PBL from untreated HD patients.

In our studies, as in those of Rotstein *et al.* (1983), most of the untreated HD patients (75%) were normal NK responders. A number of patients who showed low NK responses belonged to LD grade of the disease and clinical stage IV, indicating that deficiency in NK activity may be associated with poor prognosis. Also, it was interesting to note that, of the few patients (n = 7) who could be classified

**Figure 2** NK responder status of HD patients belonging to various histopathological grades and clinical stages.

as high NK responders (cytotoxicity more than mean percent cytotoxicity of healthy donors + 2 S.D. ≥74% Figure 1) none had stage IV or LD grade of the disease. Although the trend of observations indicated association of NK activity with stage and grade of the disease, owing to the limitation in the number of patients in these categories available for studies, no definitive conclusion can be made.

The PBL from a group of ten healthy donors and ten HD patients were phenotyped for HNK-1 (a NK cell subset marker) positivity. The percentage of HNK-1 + cells was comparable in healthy donors (8.3 ± 1.6) and HD patients (10.9 ± 2.7). This indicates that the low NK activity shown by 25% of HD patients may not correlate with the percent of cells bearing NK phenotype.

Most other investigators have reported significantly lower NK activity in active HD patients (Berenyi *et al.*, 1986; Frydecka, 1985; Hawrylowicz *et al.*, 1982; Levy *et al.*, 1984; Tursz *et al.*, 1982; Komiyama *et al.*, 1987) suggesting that the defect in the killing ability of NK cells in a child with HD could be due to a deficient release of NKCF. Normal NK activity has been demonstrated in HD patients in remission (Berenyi *et al.*, 1986).

In recent years, LAK cells have been considered an important cytotoxic effector mechanism, owing to the ubiquitous presence of LAK cell precursors, the ease with which LAK cells can be generated *in vitro* and their broad spectrum of lytic activity (Grimm, 1986; Rosenberg, 1988). LAK cell therapy has been tried in solid tumours and in NHL (Rosenberg, 1988; Rosenberg *et al.*, 1987) but not in HD.

Our data on LAK cell activity showed that the HD patients exhibited equivalent or better LAK cell activity compared with healthy donors, irrespective of the histopathological grade or clinical stage of the disease. Oshimi *et al.* (1986) have studied LAK cell generation in lymphomas. They reported that short-term culture as well as *in vitro* expanded LAK cells can efficiently kill autologous and allogeneic target lymphoma cells. Lymphoma cells of clinically high grade were shown to be more susceptible to LAK cell lysis than those of low and intermediate grades. Dawson *et al.* (1986) have also reported the relative resistance of lymphoma targets to LAK cell killing. There are no reports, so far, on the susceptibility of Reed-Sternberg cells to LAK cell lysis.

The frequency of LAK cell precursors in PBL of patients with lymphomas has not yet been reported. Here we have shown a comparable frequency of LAK cell progenitors in healthy donors and HD patients which is in line with their equivalent LAK cell function.

Therefore, our study reveals that, although HD patients show T cell hyporesponsiveness, two of the main cytotoxic effector mechanisms, namely, NK and LAK cell activities, appear to be efficient in PBL of untreated HD patients.

**Figure 3** LAK cell activity of PBL from healthy donors and patients with active HD.

**Figure 4** Frequency distribution of LAK cell precursors in PBL of healthy donors and patients with active HD.

### References

AGHAI, E. (1986). Hodgkin disease: malignancy, inflammation and abnormal immunity. *Leuk. Res.*, **10,** 1267.

BERENYI, E., SURANYI, P., PALOCZI, K. & SZEGEDI, G. (1986). Large granular lymphocytes, Leu 7 reactivity and natural killer cell function in peripheral blood of patients with Hodgkin's disease. *Cancer Immunol. Immunother.*, **21,** 164.

DABHOLKAR, M., ADVANI, S., TATAKE, R. & GANGAL, S. (1986). Natural and antibody-dependent cellular cytotoxicity in chronic myeloid leukemia patients in remission. *Leuk. Res.*, **10,** 203.

DAMLE, R.N., TATAKE, R.J., ADVANI, S.H. & GANGAL, S.G. (1990). Affinity of IL-2 receptors and proliferation of mitogen activated lymphocytes in Hodgkin's disease. *Br. J. Cancer*, **61,** 404.

DAWSON, M.M., JOHNSTON, D., TAYLOR, G.M. & MOORE, M. (1986). Lymphokine activated killing of fresh human leukemias. *Leuk. Res.*, **10,** 683.

FRYDECKA, I. (1985). Natural killer cell activity during the course of disease in patients with Hodgkin's disease. *Cancer*, **56,** 2799.

GANGAL, S.G. & MUKHOPADHYAYA, R. (1987). Interleukin-2 and its role in cancer. *Ind. J. Allergy Applied Immunol.*, **1,** 3.

GRIMM, E.A. (1986). Human lymphokine-activated killer cells (LAK) as a potential immunotherapeutic modality. *Biochim. Biophys. Acta*, **865,** 267.

GULWANI, B.N., ADVANI, S.H. & GANGAL, S.G. (1986). Functional activities of enriched Fc receptor bearing T lymphocytes in Hodgkin's disease. *Oncology*, **43**, 149.

HAWRYLOWICZ, C.M., REES, R.C., HANCOCK, B.W. & POTTER, C.W. (1982). Depressed spontaneous natural killing and interferon augmentation in patients with malignant lymphoma. *Eur. J. Cancer Clin. Oncol.*, **18**, 1081.

JOSHI, N.N., MUKHOPADHYAYA, R., ADVANI, S.H. & GANGAL, S.G. (1987). Production of Interleukin-2 and expression of Tac antigen in Hodgkin's disease. *Cancer Detect. Prevent.*, **Suppl. 1**, 137.

KARANDE, A.A., GULWANI, B., ADVANI, S.H. & GANGAL, S.G. (1982). Subpopulations of human T lymphocytes in patients with Hodgkin's disease before and after treatment. *Neoplasma*, **29**, 149.

KOMIYAMA, A., KAWAI, H., YAMADA, S. & 4 others (1987). A killing defect of NK cells with the absence of NK cytotoxic factors in a child with Hodgkin's disease. *Blood*, **69**, 1686.

LEVY, S., TEMPE, J.L., ALEKSIJEVIC, A. & 4 others (1984). Depressed NK cell activity of peripheral blood mononuclear cells in untreated Hodgkin's disease: enhancing effect of interferon *in vitro*. *Scand. J. Haematol.*, **33**, 386.

LOTZOVA, E. (1987). Interleukin-2 generated killer cells, their characterisation and role in cancer therapy. *Cancer Bull.*, **39**, 30.

LOTZOVA, E. (1985). Effector immune mechanisms in cancer. *Nat. Immun. Cell Growth Regul.*, **4**, 293.

MOGHE, M.V., ADVANI, S.H. & GANGAL, S.G. (1980). Demonstration of inhibitory factors affecting cell-mediated immunity in patients with Hodgkin's disease. *Eur. J. Cancer*, **16**, 937.

MOGHE, M.V., ADVANI, S.H. & GANGAL, S.G. (1981). Suppressive effect of adherent mononuclear cells from peripheral blood of patients with Hodgkin's disease on PHA responsiveness. *Ind. J. Exp. Biol.*, **19**, 1101.

MUKHOPADHYAYA, R., ADVANI, S.H. & GANGAL, S.G. (1983). Impairment of T lymphocyte colony formation in Hodgkin's disease: effect of soluble inhibitory factors on normal T lymphocyte colony formation potential. *Acta Haematol.*, **70**, 357.

MUKHOPADHYAYA, R., ADVANI, S.H. & GANGAL, S.G. (1987a). Functional evaluation of T lymphocytes from peripheral blood and spleens in Hodgkin's disease. *Br. J. Cancer*, **56**, 800.

MUKHOPADHYAYA, R., ADVANI, S.H. & GANGAL, S.G. (1987b). Effect of exogenous Interleukins on in vitro responses of T lymphocytes from patients with Hodgkin's disease. *Cancer Detect. Prevent.*, **10**, 445.

OSHIMI, K., OSHIMI, Y., AKUTSU, M. & 4 others (1986). Cytotoxicity of Interleukin-2 activated lymphocytes for leukemia and lymphoma cells. *Blood*, **68**, 938.

RAJARAM, N., TATAKE, R.J., ADVANI, S.H., NAIK, S.L. & GANGAL, S.G. (1990). Natural killer and lymphokine activated killer cell functions in chronic myeloid leukemia. *Cancer Immunol. Immunother.*, **31**, 44.

ROMAGNANI, S., FERRINI, P.L. & RICCI, M. (1985). The immune derangement in Hodgkin's disease. *Semin. Haematol.*, **22**, 41.

ROSENBERG, S.A. (1988). Immunotherapy of patients with advanced cancer using Interleukin-2 alone or in combination with lymphokine activated killer cells. In: *Important Advances in Oncology*, De Vita, V.T., Hellman, S. & Rosenberg, S.A. (eds) Philadelphia: J.B. Lippincott Co., p217.

ROSENBERG, S.A., LOTZE, M.T., MUUL, L.M. & 9 others (1987). A progress report on the treatment of 157 patients with advanced cancer using lymphokine activated killer cells and interleukin-2 or interleukin-2 alone. *N. Engl. J. Med.*, **316**, 889.

ROTSTEIN, S., BARAL, E., BLOMGREN, H. & JOHANSSON, B. (1983). *In vitro* radiosensitivity of the spontaneous cytotoxicity of blood lymphocytes in patients with untreated Hodgkin's disease. *Eur. J. Cancer Clin. Oncol.*, **19**, 1405.

TATAKE, R.J., KRISHNAN, N., RAO, R.S., FAKIH, A.R. & GANGAL, S.G. (1989). Lymphokine-activated killer cell function of lymphocytes from peripheral blood, regional lymph nodes and tumor tissues of patients with oral cancer. *Int. J. Cancer*, **43**, 560.

TURSZ, T., DOKHELAR, M.C., LIPINSKI, M. & AMIEL, J.L. (1982). Low NK cell activity in patients with malignant lymphoma. *Cancer*, **50**, 2333.